# Using Sewage Sludge Ash as an Efficient Adsorbent for Pb (II) and Cu (II) in Single and Binary Systems

**DOI:** 10.3390/molecules25112559

**Published:** 2020-05-31

**Authors:** Bogdan Adrian Militaru, Rodica Pode, Lavinia Lupa, Winfried Schmidt, Agnes Tekle-Röttering, Norbert Kazamer

**Affiliations:** 1 Faculty of Industrial Chemistry and Environmental, Politehnica University of Timisoara, 300006 Timisoara, Romania; rodica.pode@upt.ro (R.P.); lavinia.lupa@upt.ro (L.L.); 2 Westfälische Hochschule, University of Applied Sciences, 45897 Gelsenkirchen, Germany; winfried.schmidt@w-hs.de (W.S.); agnes.tekle-roettering@w-hs.de (A.T.-R.); norbert.kazamer@w-hs.de (N.K.)

**Keywords:** sewage sludge ash, adsorption, adsorbent, lead, copper

## Abstract

Incineration of sewage sludge produces every year huge amounts of sewage sludge ash. Due to its porosity and composition, sewage sludge ash can be used as an adsorbent for heavy metal ions removal. The present paper discusses the efficiency and feasibility of its use as an adsorbent for Pb (II) and Cu (II) removal in single and binary systems. Sewage sludge ash dosage, pH influence, equilibrium and kinetic studies were examined. The results show that sewage sludge ash is an effective and environmentally friendly adsorbent. The maximum adsorption capacity was 25.0 mg/g for Pb (II) and 7.5 mg/g for Cu (II). The presence of the competitive metal led to lower adsorption rate. The study concludes that sewage sludge ash is a promising adsorbent for Pb (II) and Cu (II) removal from wastewater presenting both economic and environmental benefits.

## 1. Introduction

The management of the sewage sludge generated by municipal wastewater plants has become an important issue in the last years [1]. 

Twenty-seven European Union countries generated more than 11.5 million tons of sewage sludge in 2015, and this quantity is expected to increase up to 13 million tons by 2020 due to industrialization and urbanization [2,3,4,5]. In the past few years, European Union promoted the ecological management of such wastes by introducing directives regarding sewage sludge management and therefore classical methods, such as storage, are being replaced by methods leading to waste stabilization and safe recycling.

There are several methods for sewage sludge management presented in the literature: anaerobic co-digestion [6], composting [7], incineration [8], co-incineration [9] and cementing [10].

Incineration is one of the most used alternatives. Even though this method reduces the volume and stabilizes the sewage sludge, its disposal still represents a significant environmental issue [11]. Therefore, another important aim is to find strategies to manage this waste. Many studies focused on using sewage sludge ash to produce construction materials [12] or to recover phosphorus [13].

Sewage sludge ash is a porous material that commonly contains silicon oxide, calcium phosphates, iron oxide and aluminum oxide. These characteristics make it a potential adsorbent for pollutant removal from aqueous solutions [14,15]. In the literature, there are some studies regarding adsorption of heavy metal ions using wastes such as display panel glasses [16], cotton [17] or fly ash [18]. Management of the waste and wastewater treatment are the benefits of using waste materials as adsorbents. There are few studies regarding the utilization of sewage sludge ash as an adsorbent. Peer reviewed literature has reported that sewage sludge ash was also used as an adsorbent for copper, zinc, cadmium [15,19] and lead removal [14].

Heavy metal ion pollution in water is an important environmental issue due to carcinogenicity and toxicity properties. Both lead and copper are metals that can endanger and poison the human body, and especially children are affected by lead poisoning [20]. Lead and copper contaminations coexist in wastewaters that come from industries such as battery manufacturing, metal plating and electronics [21]. However, to the best of our knowledge, there are no studies regarding adsorption of lead and copper simultaneously using sewage sludge ash as an adsorbent. Therefore, the purpose of this research was to study the applicability of sewage sludge ash as an adsorbent for lead and copper removal from aqueous solutions in single and binary systems by investigating the effects of pH, adsorbent dosage, contact time and initial metal concentration. Additionally, by using chemical analysis, isotherm models and kinetic models the adsorption mechanism was investigated. The proposed method represents a good alternative for the stringent legislation regarding the disposal of sewage sludge and presents a valuable option from both economic and environmental perspectives by using this waste as an efficient adsorbent for water treatment containing two important and toxic heavy metal ions.

## 2. Results and Discussion

### 2.1. Characterization of Sewage Sludge Ash

A detailed analysis of the chemical composition of the sewage sludge ash can be found in our previous reported work [22]. The main elements are Ca, Fe, P and Al. The physical properties of the sewage sludge ash are presented in Table 1.

#### XRD and SEM–EDX Analysis

**Figure 1:**
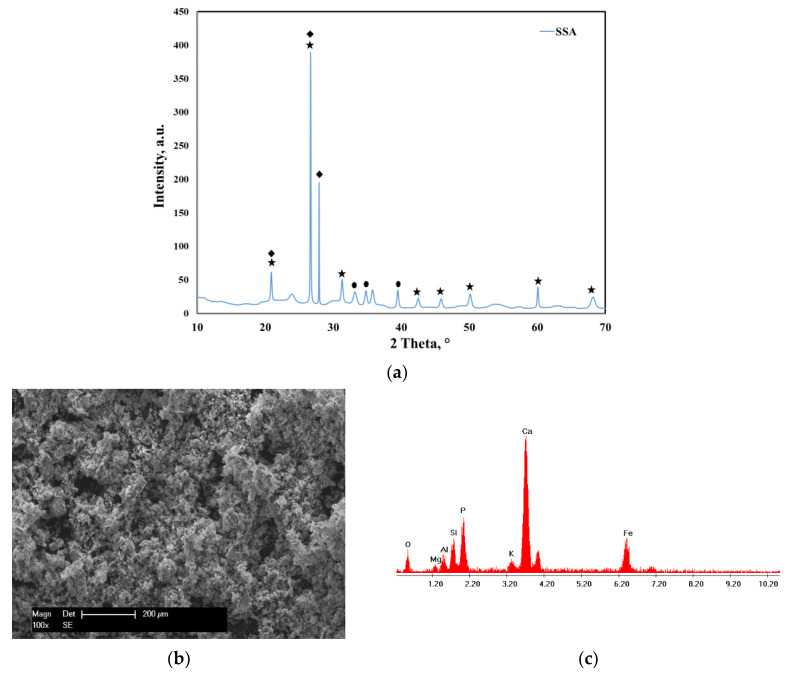
(**a**) XRD pattern of the sewage sludge ash (SSA) before adsorption: quartz, SiO_2_—COD ID: 01-075-6051, calcium phosphate, Ca_2_P_2_O_7_—COD ID: 00-045-1061, hematite, Fe_2_O_3_—COD ID: 01-076-4579; (**b**) SEM micrograph of the sewage sludge ash; (**c**) EDX spectra of the sewage sludge ash.

The XRD pattern of the sewage sludge ash (SSA) before adsorption is presented in Figure 1a. The main crystalline phases identified before adsorption revealed the presence of quartz (SiO_2_), calcium phosphate (Ca_2_P_2_O_7_) and hematite (Fe_2_O_3_).

The morphology of the SSA before adsorption was observed from the SEM image displayed in Figure 1b. It was observed that the SSA had irregular-shaped particles with different sizes. Additionally, the EDX spectra presented in Figure 1c show the presence of the main elements such as Ca, K, P, Si, Fe, Mg and Al.

### 2.2. Effect of pH

The behavior of heavy metals in solution (such as hydrolysis, complexation or precipitation) is influenced by its pH value. Additionally, the initial pH of the solutions with metal ion content plays an important role in the adsorption process due to the fact that it influences both the species of the metal present in the solution and the surface of the adsorbent. For this reason, the pH effect upon the adsorption performance of the sewage sludge ash in the removal process of lead and copper ions from aqueous solutions were discussed.

At pH higher than 7, the lead ions are efficiently removed from solution, but in this case were not removed due to an adsorption process but were removed due to precipitation process according to the potential–pH diagram for Pb ions in water systems [23,24,25,26]. Dissolved ionic species present at pH values higher than 7 are Pb(OH)^+^ (aq), Pb_4_(OH)_4_^4+^ (aq) and Pb(OH)_2_. Cu^2+^ is a copper predominant species at pH < 5.00, and for pH values higher than 5.00, species such as CuOH^+^ (aq), [Cu_2_(OH)_2_]^2+^ (aq) and Cu(OH)_2_ (aq) are present [15,26]. The variation in pH between 2 and 7 avoided the precipitation of Pb (II) and Cu (II).

**Figure 2:**
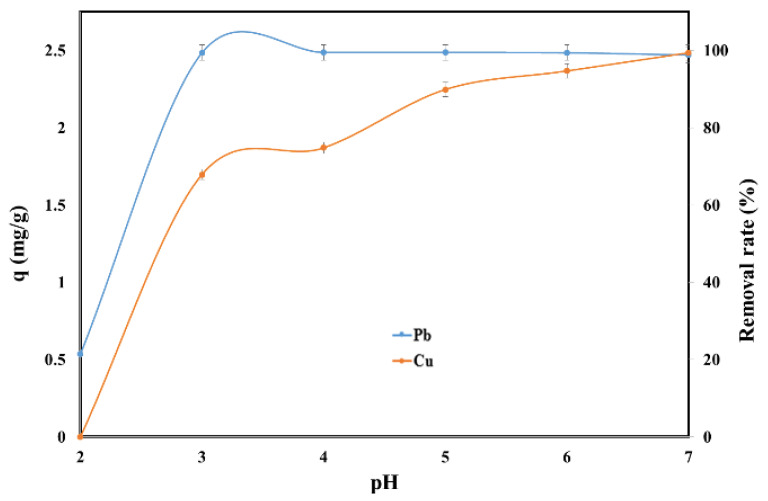
Effect of pH on the adsorption of Pb (II) and Cu (II) by SSA.

The pH has a strong influence on the sorption of Pb (II) and Cu (II) on the SSA because H^+^ ions can compete adsorption sites with Pb^2+^ and Cu^2+^. The effect of the initial pH on the adsorption capacity and removal rate of Pb (II) and Cu (II) in single system is presented in Figure 2.

The adsorption of Cu (II) at pH = 2 was negligible while for Pb (II) the adsorption capacity was 0.53 mg/g SSA. The adsorption capacity increased for both of Pb (II) and Cu (II) with the increase of the initial pH. For Pb (II) the plateau was reached after pH = 3 when the adsorption capacity was 2.49 mg/g SSA. On the other hand, the adsorption capacity for Cu (II) continued to increase with the increase of the initial pH reaching the maximum of 2.49 mg/g SSA at pH = 7. Therefore, the pH = 6 was considered to deliver the best performance for both metals. Similar results were obtained when the polypyrrole-based activated carbon, sawdust and rice husks were used as adsorbents for the removal of Pb, and the maximum adsorption capacity occurring at pH = 5–6.5 [27,28,29]. Additionally, the removal of copper took place at an initial pH value close to 6 when bottom ash of expired drug incineration, and shells of lentil, wheat and rice were used as adsorbents [30,31,32].

### 2.3. Effect of Adsorbent Dosage

**Figure 3:**
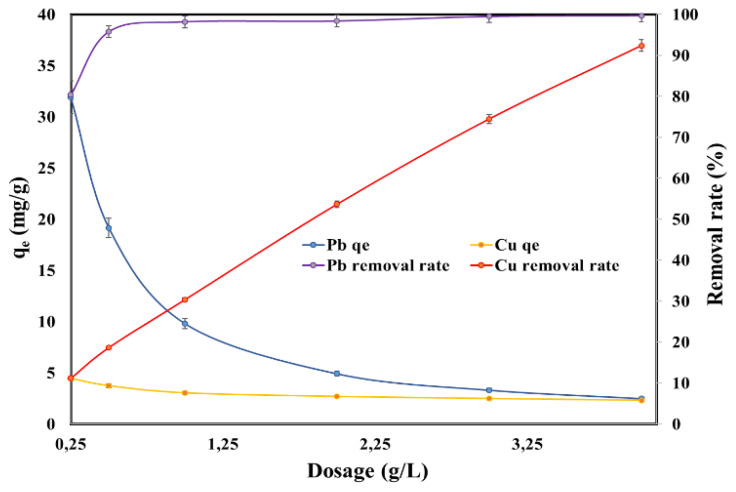
Effect of adsorbent dosage on the adsorption capacity and on the removal rate of Pb (II) and Cu (II).

Figure 3 displays the influence of SSA dosage on the adsorption capacity and on the removal rate of Pb (II) and Cu (II). The adsorption capacity of the heavy metal ions decreased while the removal rate increased with the increasing SSA dosage. It can be noticed that the maximum adsorption capacity was considerably higher for Pb (II) (31.9 mg/g) than for Cu (II) (4.44 mg/g). For Pb (II), the removal rate increased from 80.4% to 96% when the SSA dosage increased from 0.25 g/L to 0.5 g/L, and thereafter the removal rate of Pb (II) reached the plateau and the removal rate was almost 100%. For Cu (II), on the other hand, the removal rate increased constantly with the increasing of dosage from 11.2% to 92.4% when the SSA dosage was increased from 0.25 g/L to 4 g/L.

### 2.4. Kinetic Studies

**Figure 4:**
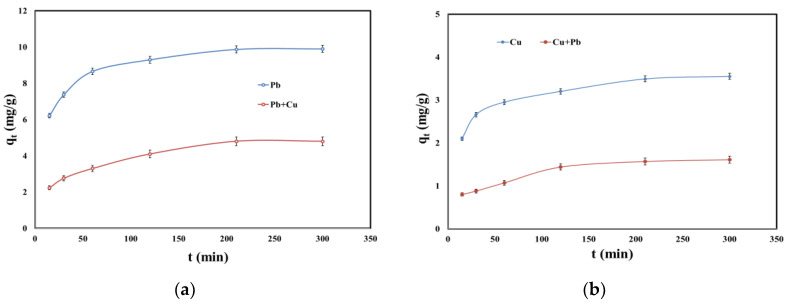
(**a**) Effect of contact time on the adsorption of Pb (II) and Pb (II) in the presence of Cu (II); (**b**) effect of contact time on the adsorption of Cu (II) and Cu (II) in the presence of Pb (II).

Kinetic studies were used to determine the optimum necessary time to establish the equilibrium between SSA and Pb (II) and Cu (II) in single and binary systems. The effect of contact time on the adsorption of Pb (II) and Cu (II) in single and binary systems is presented in Figure 4.

The adsorption process occurred already in the first minutes of contact between SSA and both metals in single and binary systems. After 210 min, the adsorption capacity rate decreased. Comparing the adsorption in single and binary systems, the latter one became slower in the presence of the competitive metal ion. For example, the adsorption capacity for Pb (II) in single system at 210 min was 9.87 mg/g, and the adsorption capacity for Pb (II) in the presence of Cu (II) dropped to 4.80 mg/g. Additionally, the adsorption capacity for Cu (II) in single system at 210 min was 3.49 mg/g and dropped to 1.57 mg/g in the presence of Pb (II).

**Figure 5:**
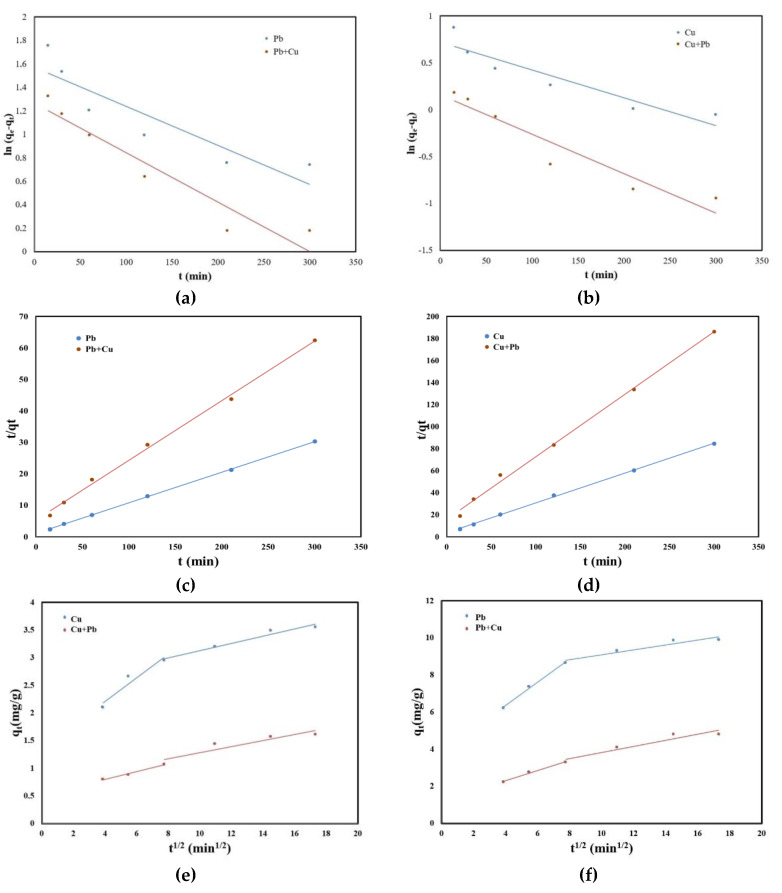
(**a**) Pseudo-first-order kinetic model of Pb (II) and Pb (II) in the presence of Cu (II) adsorption; (**b**) pseudo-first-order kinetic model of Cu (II) and Cu (II) in the presence of Pb (II) adsorption; (**c**) pseudo-second-order kinetic model of Pb (II) and Pb (II) in the presence of Cu (II) adsorption; (**d**) pseudo-second-order kinetic model of Cu (II) and Cu (II) in the presence of Pb (II) adsorption; (**e**) intraparticle diffusion model of Pb (II) and Pb (II) in the presence of Cu (II) adsorption; (**f**) intraparticle diffusion model of Cu (II) and Cu (II) in the presence of Pb (II) adsorption.

The adsorption kinetics that describe the removal rate of the studied metal ions is an important feature that defines the efficiency of the adsorption process. In order to determine the mechanism of adsorption of metal ions on the studied material, the experimental data were processed using the pseudo-order kinetic model one, the pseudo-order kinetic model and intraparticle diffusion model. The kinetic models used to stimulate the kinetics of Pb (II) and Cu (II) in single and binary systems on the SSA are presented in Figure 5.

The kinetic parameters together with the obtained correlation coefficients are presented in Table 2. The results showed that the values of correlation coefficient for both single and binary systems were higher for the pseudo-second-order kinetic model than those obtained for pseudo-first-order kinetic model. Additionally, the calculated adsorption capacities for the pseudo-second-order kinetic model remained similar to the experimentally reported ones.

Following the analysis of the plots according to the intraparticle diffusion kinetic model (Figure 5e,f, it can be observed that the straight line does not pass through origin. Therefore, the rate-limiting step for Pb (II) and Cu (II) adsorption is not the intraparticle diffusion model. Additionally, the line presents a discontinuity after a while, suggesting that the adsorption process is more complex. The fast adsorption of Pb (II) and Cu (II) in the first minutes was due to the film diffusion when the sewage sludge ash surface was covered by Pu (II) and Cu (II) ions. The second straight line obtained through the representation of q_t_ versus t^1/2^ suggested that after the surface coverage, the transport of Pb (II) and Cu (II) ions inside the sewage sludge ash occurred [33].

Therefore, the pseudo-second-order kinetic model can be used to simulate the experimental data regarding Pb (II) and Cu (II) adsorption in single and binary systems using SSA as an adsorbent. Nevertheless, this means that the process is controlled by chemical sorption [34,35].

### 2.5. Equilibrium Studies

**Figure 6:**
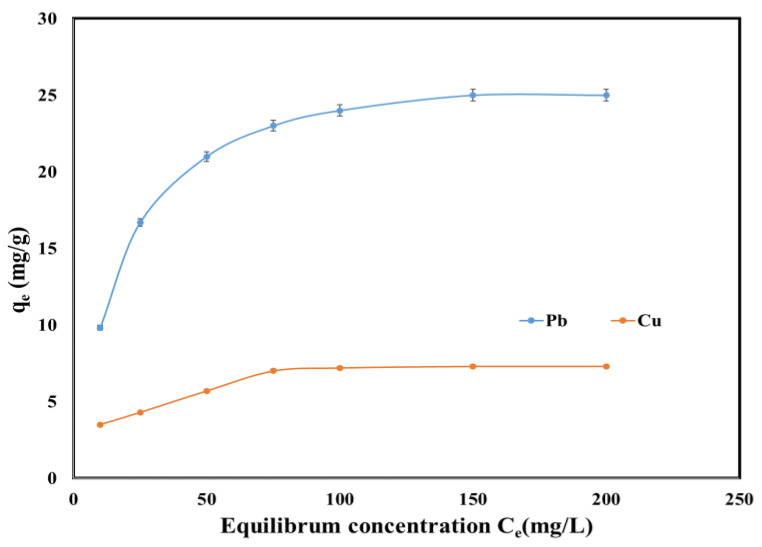
Equilibrium isotherm of Pb (II) and Cu (II) onto SSA.

Figure 6 displays the dependence of the adsorption capacities as a function of the Pb (II) and Cu (II) concentrations at equilibrium. Increasing the initial concentration of Pb (II) and Cu (II) increased the active sites available, and therefore the adsorption capacity increased. The maximum q_e_ during Pb (II) and Cu (II) removal was 25 mg/g and 7.5 mg/g, respectively.

In order to achieve the design of this adsorption study, the maximum adsorption capacity of SSA, the equilibrium coefficient and the adsorption mechanism must be considered. Therefore, several isotherms in their linear form were studied: Langmuir, Freundlich, Temkin and Dubinin–Radushkevich (DR). The Langmuir isotherm states that the adsorption process takes place in a single layer on a uniform surface and equivalent sites of the adsorbent. The Freundlich isotherm expresses the affinity of the adsorbent to the metals, while the Temkin isotherm states that the surface of the adsorbent is heterogenous.

**Figure 7:**
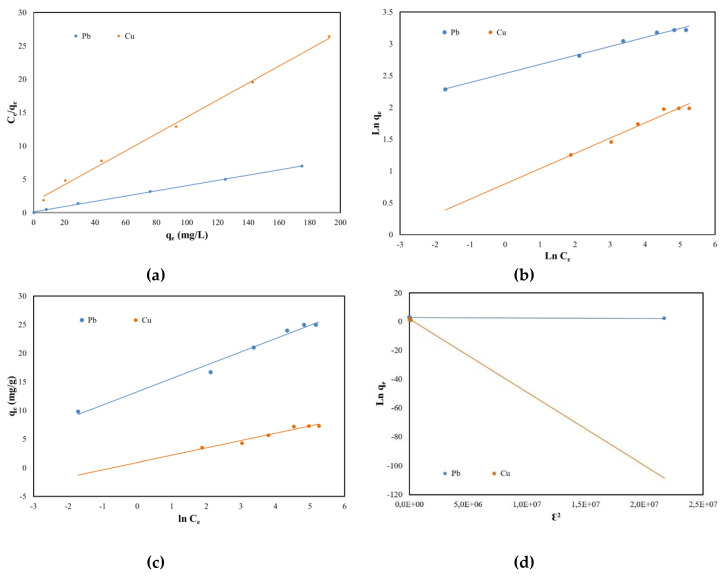
Equilibrium isotherms of Pb (II) and Cu (II) adsorption onto SSA: (**a**) Langmuir; (**b**) Freundlich; (**c**) Temkin and (**d**) Dubinin–Radushkevich.

In order to design the equilibrium adsorption regarding Pb (II) and Cu (II) removal using sewage sludge ash as an adsorbent, the linear graphs and the equilibrium sorption isotherms were plotted and can be consulted in Figure 7 and Table 3 respectively. By comparing the results from Table 3 it can be concluded that both Pb (II) and Cu (II) removal from aqueous solutions occurred as a monolayer at the uniform surfaces of the SSA because the Langmuir isotherm obtained the highest regression coefficients (0.999 for Pb (II) and 0.998 for Cu (II)). Further, there was no significant difference between the maximum adsorption capacity determined experimentally and those calculated from its plot for both Pb (II) and Cu (II). The lowest regression coefficients were obtained for DR isotherms. Likewise, a difference for the qs parameter can be as well observed when comparing the values of the theoretically determined to the ones experimentally obtained.

### 2.6. Adsorption Mechanism and Performance

**Figure 8:**
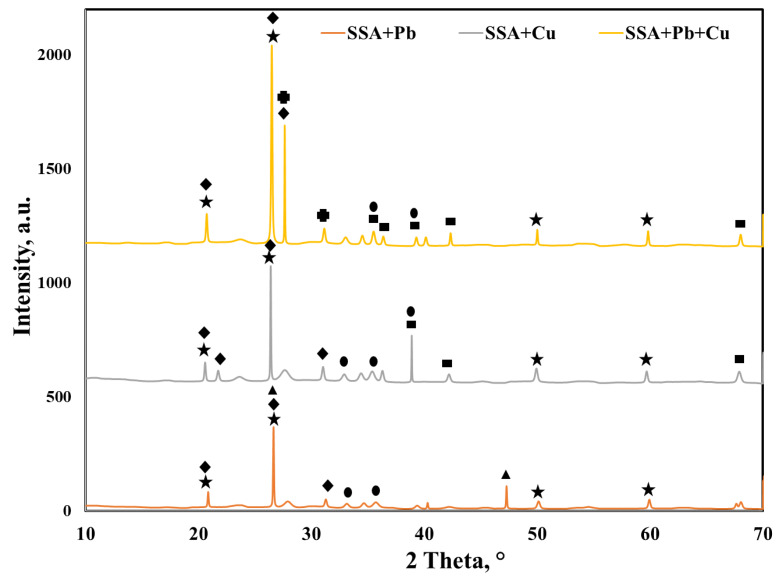
XRD patterns of the SSA after adsorption of heavy metal ions in single and binary systems: 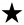 quartz, SiO_2_—COD ID: 01-075-6051, 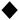 calcium phosphate, Ca_2_P_2_O_7_—COD ID: 00-045-1061, 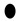 hematite, Fe_2_O_3_—COD ID: 01-076-4579, 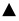 lead phosphate, Pb_2_P_2_O_7_—COD ID: 00-013-0273, 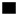 copper oxide, CuO—COD ID: 00-03-0884, 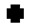 lead silicate, Pb_2_SiO_4_—COD ID: 00-037-0203.

The overlapped XRD spectra of the SSA after Pb (II), Cu (II) and binary adsorption presented in Figure 8 show the formation of new crystalline phases. After Pb (II) and Cu (II) adsorption, quartz and hematite remained in the SSA. Calcium phosphate was also present in the SSA, yet the peak at 2θ = 28° could not be identified anymore.

Furthermore, after Pb (II) adsorption, new peaks associated with lead phosphate (Pb_2_P_2_O_7_) could be identified. After Cu (II) adsorption, new peaks associated with copper oxide (CuO) could be detected. The binary system adsorption led to the identification of the lead silicate (Pb_2_SiO_4_) and copper oxide (CuO) peaks. These new crystalline phases identified by XRD confirmed that chemical reactions between SSA and Peb (II) and Cu (II) took place and also suggested that Ca–P bound in the SSA was partially broken.

Wang et al. [14] studied the adsorption of lead using incinerated sewage sludge ash as an adsorbent and identified new crystalline phases, suggesting the precipitation of lead as PbSO_4_. Nevertheless, the adsorption of copper in single and binary systems (Cu–Zn, Cu–Cd) [15] did not show any new crystalline phases.

**Figure 9:**
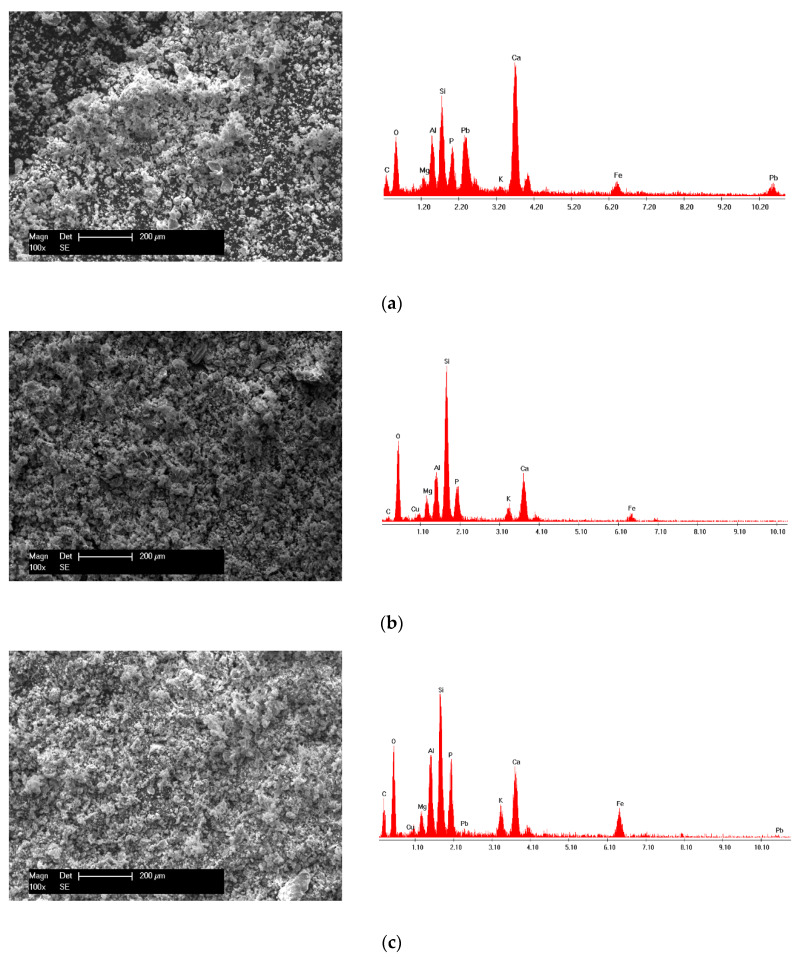
SEM micrographs and EDX spectra of the (**a**) SSA after Pb (II) adsorption; (**b**) SSA after Cu (II) adsorption and (**c**) SSA after both Pb (II) and Cu (II) adsorption.

The micrographs displayed in Figure 9 show a topographical change following the adsorption of Pb (II) and Cu (II) in both single and binary systems. The surface was much denser, and uniformly sized crystalline phases were formed as agglomerates in a much more regular way. Additionally, the EDX spectra presented in Figure 9 shows the presence of Pb and Cu after adsorption on the surface of the SSA.

The adsorption capacity and concentrations of Ca, K and Mg after sorption experiments for both Pb (II) and Cu (II) for 10, 75 and 150 mg/L are presented in Table 4. The adsorption capacity of Pb (II) was considerably higher than for Cu (II) due to the electronegativity of Pb^2+^ and Cu^2+^. Stronger attraction occurred between adsorbent and metal ion with higher electronegativity values (Pb: 2.33 and Cu: 1.95) [36]. Additionally, the smaller the hydrated ionic radius, the greater the affinity of the metal for the adsorption process (Pb: 4.01 Å and Cu: 4.19 Å) [37]. Therefore, it can be stated that the adsorption affinity of Pb (II) is higher than that of Cu (II).

Additionally, another important aspect in order to evaluate the adsorption mechanism is to note that higher adsorption capacities and higher concentrations of Pb (II) and Cu (II) led to higher concentrations of Ca, Mg and K in solutions after sorption experiments. This suggests that the adsorption mechanism of Pb (II) and Cu (II) by SSA is associated with cationic exchange. When the Cu (II) concentration was increased from 10 mg/L to 150 mg/L, the concentrations of Ca, K and Mg increased from 4.80 mg/L, 0.470 mg/L and 0.190 mg/L to 4.92 mg/L, 0.580 mg/L and 0.240 mg/L, respectively. For the same initial concentration of Pb (II) and Cu (II), the adsorption capacity and Ca, K and Mg concentration at equilibrium were higher for Pb (II).

**Figure 10:**
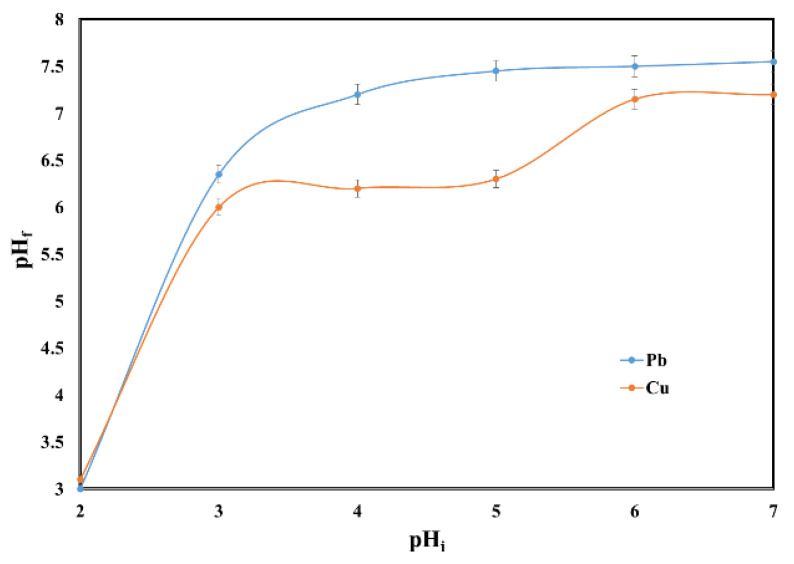
Effect of initial pH on the final pH.

Higher concentration of cations (Ca^2+^, Mg^2+^, K^+^) in solution after sorption experiments led to higher values of final pH (Figure 10). The final pH value at the end of the process led to an increase from 3 to 7.55 for Pb (II) and from 3.1 to 7.2 for Cu (II). The alkali properties of the sewage sludge ash and the neutralization effect on aqueous solutions could be noticed.

Correlating the results from RX diffractograms, SEM images and EDX spectra with the changes in final pH and in cation concentrations in solution after sorption experiments, it can be concluded that the heavy metal ion adsorption process onto SSA is controlled by chemisorption involving cation exchange.

Likewise, the pseudo-second-order kinetic model indicates that the process is controlled by chemisorption involving cation exchange. Wang et al. [15] also indicated that the primary adsorption mechanism of the studied heavy metal ions (Cu, Zn and Cd) by the incinerated SSA (ISSA) was cation exchange.

In order to determine if the SSA is a reliable adsorbent for Pb (II) and Cu (II) removal and to evaluate the potential environmental risk, due to the presence of metals in the sewage sludge ash [22], the influence of pH on the metal leaching of the SSA was investigated. Table 5 presents the results regarding the leaching of metals from SSA under different values of pH. As expected, the lower the pH was, the higher was the concentration of metals leached. For pH = 2, the concentrations of Zn, Mn and Al were 0.98 mg/L, 3.41 mg/L and 13.6 mg/L, respectively. For pH = 3, the concentration of Zn was 0.18 mg/L, and for Mn it was 0.27 mg/L. As the pH increased, the concentrations of leached metals decreased to values lower than 0.15 mg/L. At pH = 6, the amount of metals leached from SSA was negligible. Therefore, the SSA is a reliable adsorbent with low environmental risk.

The adsorption performances of the sewage sludge ash in the removal of process of Pb (II) and Cu (II) from aqueous solutions were compared with the adsorption capacities developed by other wastes reported in the literature. The comparison of the values (Table 6) show that the sewage sludge ash can be efficiently used as a high performance adsorbent for wastewater treatment.

## 3. Materials and Methods

### 3.1. Materials

The raw sewage sludge was collected from a municipal wastewater treatment plant from Timis county, Romania. Prior to the process of obtaining the ash, the collected sludge was dried at 105 °C for 20 h. Further, the dried sewage sludge was calcinated at 850 °C for 3 h using a Nabertherm B180 furnace.

All chemicals and reagents used were of analytical grade. Stock solutions (100 mg/L) of Pb (II) and Cu (II) were prepared by dissolving Pb(CH_3_COO)_2_ and CuSO_4_, respectively. Deionized water was used for the analytical method. Hydrochloric acid (HCl) and sodium hydroxide (NaOH) were used for pH adjustment.

### 3.2. Heavy Metal Ion Adsorption

The adsorption performance of the sewage sludge ash was studied taking into account the influence of the pH, adsorbent dosage, stirring time and initial concentration of Pb (II) and Cu (II).

The experiments were performed by mixing certain amounts of sewage sludge ash with 25 mL aqueous Pb (II) and Cu (II) solutions. The adsorption process was conducted in batch mode using Thermo Scientific Variomag Telesystem equipment for sample shaking at a constant rotation speed of 570 rpm. Further, the samples were centrifuged for measurement of metal concentrations by microwave plasma-atomic emission spectrometer (4210 MP-AES, Agilent Technologies, Melbourne, Australia). The pH was measured using a Metrohm 620 pH-meter (Herisau, Switzerland).

To examine the pH effect on the adsorption process, the experiments were conducted by adding 0.1 g of sewage sludge ash into 25 mL Pb (II) and Cu (II) solutions of 10 mg/L for 210 min. The initial pH of the solutions varied between 2 and 7. Additionally, in order to evaluate the potential environmental risk, for the same range of pH, concentrations of Zn, Ni, Co, Mn, Cr, Al and the final pH were determined.

The effect of the adsorbent dosage was studied by varying the solid:liquid ratio (0.25:1–4:1 g:L) of Pb (II) and Cu (II) solutions (10 mg/L) for 210 min at pH = 6.

Kinetic experiments were also conducted in order to evaluate the adsorbent performance in both single and binary systems. The amount of 0.025 g of sewage sludge ash (solid:liquid ration of 1:1 g:L) was added into 25 mL Pb (II) and Cu (II) solutions of 10 mg/L, at pH = 6 for certain amount of time (5–300 min). Additionally, to investigate the possible competition and to evaluate the adsorption process in the presence of both Pb and Cu, 0.025 g of sewage sludge ash was added into 25 mL Pb (II)+ Cu (II) solutions of 10 mg/L each, at pH = 6 for a time varying between 5 and 300 min.

The effect of initial concentrations was studied by mixing 25 mL of certain concentrations (10–200 mg/L) of each metal with 0.025 g of sewage sludge ash (solid: liquid ratio of 1:1 g: L) at pH = 6 for 210 min.

Equation (1) was used for the calculation of Pb (II) and Cu (II) uptake on 1 g of adsorbent.

(1) $$q_{e}=\frac{\left(C_{0}-C_{e}\right)\times V}{m}$$

where *q_e_* is the adsorbed quantity of Bb (II) and Cu (II) expressed in milligrams per 1 g of studied adsorbent; *C_0_* and *C_e_* respectively represent the Pb (II) and Cu (II) concentration of the aqueous solutions before adsorption and after the established equilibrium (mg/L); *V* is the volume of the Pb (II) and Cu (II)-containing aqueous solution (L); and *m* is the mass of the sewage sludge ash (g) used in the experiments.

### 3.3. Adsorbent Characterization

The sewage sludge ash was characterized before and after heavy metal ion adsorption by X-ray diffraction (XRD), scanning electron microscopy (SEM) and X-ray dispersion analysis (EDX). The X-ray diffraction (XRD) patterns were recorded using a Philips X‘Pert instrument having a CuKα cathode at 45 kV and 40 mA. Scanning electron microscopy (SEM) images were acquired using Philips XL 30 ESEM equipment at 25 kV and 10 mm working distance. The chemical composition of the materials was determined with the FEI energy dispersive X-ray (EDX) spectroscopy detector of the microscope.

A Micromeritics ASAP 2020 instrument was used in order to determine the specific surface area, pore volume and adsorption average pore diameter of the ash.

## 4. Conclusions

This paper reports new insights of SSA as a low-cost, efficient and environmentally friendly adsorbent for Pb (II) and Cu (II) removal in single and binary systems. The pH value played a crucial role, the best performances being obtained at pH = 6 for both metals. The amount of metals leached from SSA was negligible at pH = 6, and therefore SSA has low environmental risk. The Langmuir isotherms best described the adsorption process with the maximum capacity of 25 mg/g for Pb (II) and 7.5 mg/g for Cu (II). The presence of competitive metals in binary systems led to lower adsorption capacities for the target metal. A pseudo-second-order kinetic model was found as the best kinetic model. Correlating the experimental results, it can be concluded that the main adsorption mechanism regarding the adsorption of Pb (II) and Cu (II) using SSA as an adsorbent is cation exchange.

Using SSA as an adsorbent presents multiple benefits:

- capitalization of waste and therefore reduction of waste disposal;
- low-cost and efficiency;
- alkaline effects.

## Figures and Tables

**Table 1:** Physical properties of the sewage sludge ash.

Parameters	Values
BET surface area (m^2^/g)	7.24
Adsorption average pore diameter (nm)	17.2
Pore volume (cm^3^/g)	0.029

**Table 2:** Kinetic model parameters for Pb (II) and Cu (II) adsorption in single and binary systems.

Model	Parameter	Pb	Pb + Cu	Cu	Cu + Pb
Pseudo-first-order	K1, min^–1^	0.003	0.004	0.003	0.004
	qe calc, mg/g	4.81	3.55	2.05	1.17
	qe exp, mg/g	9.90	4.80	3.55	1.61
	R2	0.814	0.916	0.869	0.910
Pseudo-second-order	K2, min/(mg/g)	0.087	0.007	0.020	0.02
	qe calc, mg/g	10.3	5.27	3.69	1.76
	qe exp, mg/g	9.90	4.80	3.55	1.61
	R2	0.999	0.997	0.999	0.996
Intraparticle diffusion	Kint	0.628	0.274	0.213	0.071
	R2	0.995	0.991	0.924	0.983

**Table 3:** Equilibrium sorption isotherm parameters for Pb (II) and Cu (II) onto SSA.

Type of Isotherm	Parameter	Pb (II)	Cu (II)
Langmuir	K_L_, L/mg	0.280	0.079
	q_Lcalc_, mg/g	25.5	7.86
	R^2^	0.999	0.998
Freundlich	K_F_, mg/g	12.6	2.21
	1/n	0.142	0.240
	R^2^	0.993	0.962
Temkin	K_T_, L/g	299	2.06
	b_T_, J/mol	1066	1949
	R^2^	0.982	0.955
Dubinin–Radushkevich	K_ad_, mol^2^/kJ^2^	4 × 10^−8^	5 × 10^−6^
	q_s_, mg/g	22.1	6.36
	R^2^	0.825	0.658

**Table 4:** Adsorption capacity and concentrations of Ca, K and Mg of contacted solutions after sorption experiments.

Metal	Pb	Cu	Pb	Cu	Pb	Cu
C_e_ (mg/L)	10		75		150	
q_e_ (mg/g)	9.63	3.50	19	6.00	25	7.30
Ca (mg/L)	6.20	4.42	6.24	4.48	6.58	4.92
K (mg/L)	0.565	0.470	0.795	0.520	0.801	0.580
Mg(mg/L)	0.720	0.190	0.700	0.220	0.740	0.240

**Table 5:** Effect of pH on metal leaching.

Initial pH	Zn (mg/L)	Ni (mg/L)	Co (mg/L)	Mn (mg/L)	Cr (mg/L)	Al (mg/L)
7	UDL	UDL	0.02	0.07	0.06	0.11
6	UDL	UDL	0.02	0.08	0.06	0.11
5	UDL	UDL	0.02	0.08	0.06	0.06
4	UDL	UDL	0.03	0.09	0.06	0.07
3	0.18	UDL	0.02	0.27	0.06	0.1
2	0.98	UDL	0.01	3.41	0.06	13.6

UDL = under detection limit.

**Table 6:** The comparison between the adsorption capacities of other wastes used as adsorbents for Pb (II) and Cu (II) removal.

Pb Removal			Cu Removal		
Adsorbent	q_e_ (mg/g)	References	Adsorbent	q_e_ (mg/g)	References
SSA, Hong Kong, China	62.4	[14]	SSA, Hong Kong, China	7.62	[15]
Fly ash	2.5	[38]	SSA	4.10	[19]
Ash of rice husk	10.86	[39]	Fly ash	4.20	[40]
Egg shells	4.33	[41]	Egg shells	3.54	[41]
SSA, Timis County, Romania	25.0	Present paper	SSA, Timis County, Romania	7.50	Present paper
